# Construction and Analysis of Protein-Protein Interaction Network of Heroin Use Disorder

**DOI:** 10.1038/s41598-019-41552-z

**Published:** 2019-03-21

**Authors:** Shaw-Ji Chen, Ding-Lieh Liao, Chia-Hsiang Chen, Tse-Yi Wang, Kuang-Chi Chen

**Affiliations:** 1 Department of Medicine, Mackay Medical College, New Taipei City, Taiwan; 2 Department of Psychiatry, Mackay Memorial Hospital, Taitung Branch, Taiwan; 3 Bali Psychiatric Center, Department of Health, Executive Yuan, New Taipei, Taiwan; 4 Department of Psychiatry, Chang Gung Memorial Hospital at Linkou and Chang Gung University School of Medicine, Taoyuan, Taiwan; 5 Department of Medical Informatics, Tzu Chi University, Hualien, Taiwan

## Abstract

Heroin use disorder (HUD) is a complex disease resulting from interactions among genetic and other factors (e.g., environmental factors). The mechanism of HUD development remains unknown. Newly developed network medicine tools provide a platform for exploring complex diseases at the system level. This study proposes that protein–protein interactions (PPIs), particularly those among proteins encoded by casual or susceptibility genes, are extremely crucial for HUD development. The giant component of our constructed PPI network comprised 111 nodes with 553 edges, including 16 proteins with large degree (*k*) or high betweenness centrality (BC), which were further identified as the backbone of the network. JUN with the largest degree was suggested to be central to the PPI network associated with HUD. Moreover, PCK1 with the highest BC and MAPK14 with the secondary largest degree and 9^th^ highest BC might be involved in the development HUD and other substance diseases.

## Introduction

Heroin was originally synthesized in the late nineteenth century. Abstaining from heroin use is difficult, and it leads to a high relapse rate among past users^1,2^. New heroin users easily become addicted to the drug, tend to have serious withdrawal symptoms, and finally develop heroin use disorder (HUD). It has recently become a serious problem in South and East Asia^3–5^, and heroin users have the highest mortality rate among all the users of addictive substances in Taiwan^6^.

Similar to the other addictive disorders, HUD is a complex disorder resulting from the interplay between the environmental factors and the genetic predisposing factors^7–10^. Studies have suggested that HUD is a polygenic disorder and identified various susceptibility genes contributing to HUD through different mechanisms at different stages of HUD development^8,11,12^. However, the pathogenesis remains unclear. Previous studies evaluated the genetic influence on HUD rather than gene expression at the protein level. In addition to the genetic influence engendered by DNA, all biochemical processes are controlled by proteins. We propose that protein–protein interactions (PPIs), particularly those among proteins encoded by the aforementioned casual or susceptibility genes, are essential for HUD development.

In this study, we used the relevant gene biosignatures as the seeds to construct the PPI network associated with the development of HUD. The regulatory pathways were explored through topological analysis of the PPI network for the further understanding of the HUD mechanism. In a PPI network, nodes represent proteins, and edges represent interactions^13^. According to graph theory, the topological structure of the PPI network provides basic and direct information regarding the network and is associated with biological functions^14^. The combination of the topological structure of the PPI network with the relevant biological knowledge provides a promising tool for understanding the biological mechanisms of species.

Recently, topological analyses have been applied to molecular networks including protein interaction networks^13–19^, gene regulatory networks^20–22^, and metabolic networks^23–26^. Connectivity degree (*k*), betweenness centrality (BC), closeness centrality (CC), eigenvector centrality (EC), and eccentricity are the fundamental measures of nodes in network theory^27,28^. In a PPI network, nodes with large degree, defined as hub proteins, are crucial proteins, because they might be corresponding to the disease-causing genes^15,29^, whereas nodes with high BC, defined as bottlenecks, tend to indicate essential genes since they can be analogized to heavily used intersection in major highways or bridges^14,30,31^. In this study, we mainly focused on the hubs or bottlenecks that were central to the PPI network, identified the proteins with large degree or high BC as the key proteins, and considered the sub-network of these key proteins as the backbone worth further investigating the signaling pathways involved in HUD development.

Drug addiction is a psychiatric disorder resulting in maladaptive neuroplasticity that underlies the development of compulsive drug seeking and vulnerability to relapse during periods of attempted abstinence^32^. MiRNAs are small non-coding RNAs (18–25 nucleotides) that post- transcriptionally modulated gene expression by either repressing translation or inducing degradation of mRNA^33,34^. Recent studies indicate that miRNAs are considered to be ‘master regulators’ of gene expression, and they control the translation of target mRNAs, thereby regulating critical aspects of neurogenesis, synaptic plasticity and neurological disorders^35–38^. Consequently, discovering miRNA-disease association makes an important contribution to understanding the pathogenesis of diseases as well as designing diagnostic and therapeutic approaches for diseases^39–42^.

## Materials and Methods

### The identification of the susceptibility genes associated with HUD

We have identified the susceptibility genes associated with HUD in our previous case-control studies^43,44^ that included 124 Han Chinese men from Taiwan as the cases fulfilling the diagnostic criteria of HUD according to the Diagnostic and Statistical Manual of Mental Disorders, 5th edition (DSM-5), and 124 demographically similar patients as the controls getting regular medical checkups at a local medical center. The details of the 248 participants and the investigating methods are in the previous studies^43,44^. Briefly, we recruited no controls with substance-related disorders or substance use disorders except nicotine, and no participants with the other psychiatric diagnoses such as neurodevelopmental disorders, schizophrenia spectrum disorders, bipolar-related disorders, depressive disorders, neurocognitive disorders, etc.

From each participant, a total of 8 mL of venous blood was collected to establish the lymphoblastoid cell lines (LCLs) for RNA extraction and real-time quantitative PCR (qPCR) analyses. The study protocol was approved by the Ethical Committee of Bali Psychiatric Center (approval number: IRB970609-03), the written informed consents were obtained from the participants after full explanation of the protocol, and we performed all methods in accordance with the relevant guidelines and regulations. The identified results of the susceptibility genes associated with HUD were AUTS2, CD74, CEBPB, CEBPG, ENO2, HAT1, IMPDH2, JUN, MBD1, PDK1, PRKCB, RASA1, RGS3 (listed in Table S1), and we considered them as the seed proteins to construct the PPI network associated with HUD.

### The construction of the PPI network associated with HUD

**Figure 1:**
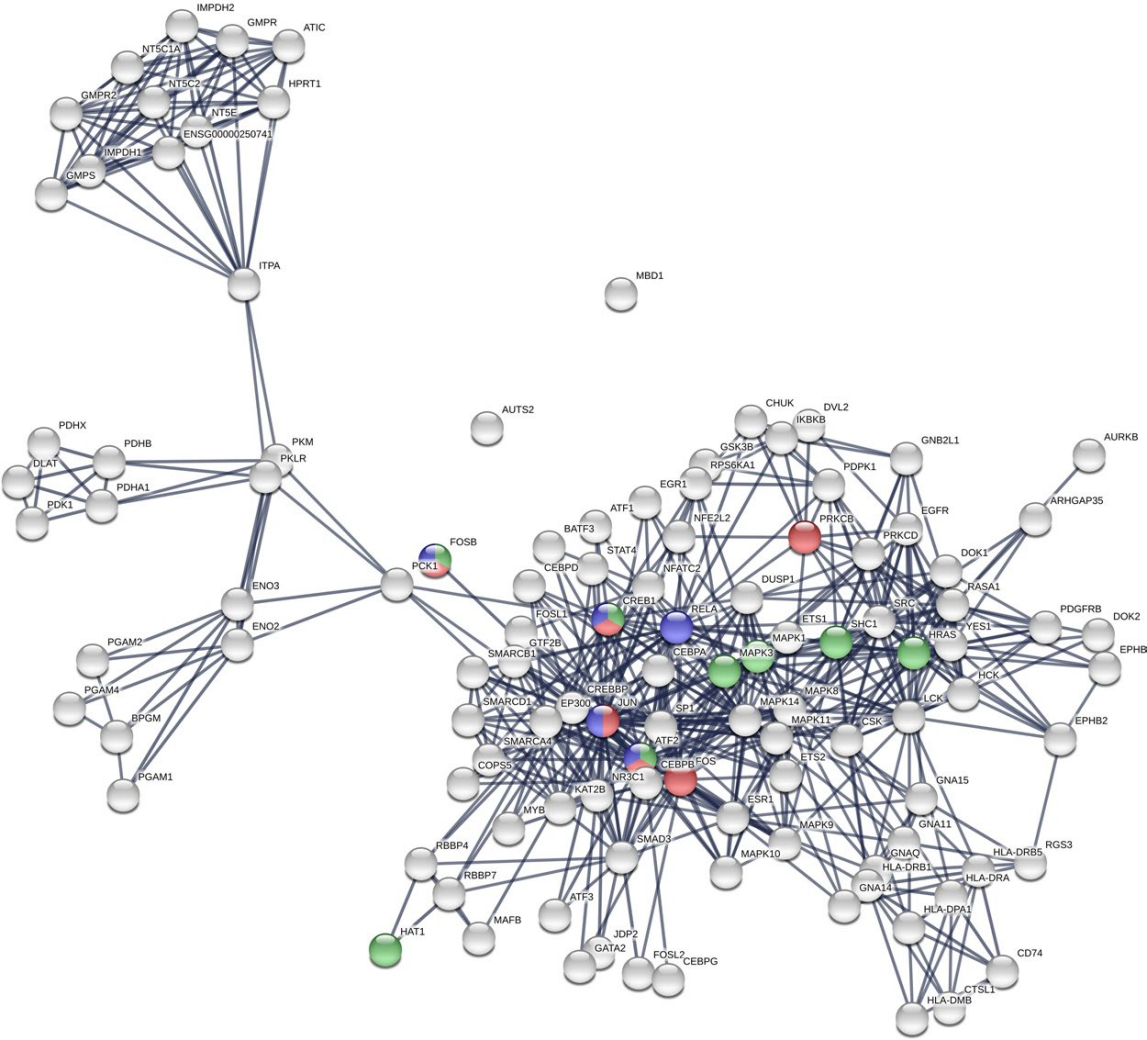
The PPI network.

We used the Search Tool for the Retrieval of Interacting Genes/Proteins database (STRING v10.5)^45^ to construct the PPI network associated with HUD. Given a list of the proteins as input, STRING can search for their neighbor interactors, the proteins that have direct interactions with the inputted proteins; then STRING can generate the PPI network consisting of all these proteins and all the interactions between them. Based on the seed proteins as input, we first constructed the PPI network (Fig. 1) associated with HUD containing the seed proteins and their neighbors. All the interactions between them were derived from high-throughput lab experiments and previous knowledge in curated databases at high level of confidence (sources: experiments, databases; score ≥ 0.90).

We further searched for the interactions derived from three sources, lab experiments, curated databases, and gene expression data, with the same confidence to construct the PPI network with the co-expression interactions (Fig. S1) for comparison. In addition, Gephi^46^, a program for large network analysis, was used to analyze the structure of the PPI networks.

### The topological analysis of the PPI network

To evaluate the nodes in the PPI networks, we adopted several topological measures including degree (*k*), between centrality (BC), eccentricity, closeness centrality (CC), eigenvector centrality (EC), and clustering coefficient^47,48^. The first two measures, degree (*k*) and BC, are often used for detecting the hub or bottleneck in a network. Degree (*k*) of a node is defined as the number of edges linked to it. A node with high degree (*k*) denotes a hub having many neighbors. BC of a node is the proportion of the number of shortest paths passing through it to the number of all the shortest paths in the network, quantifying how often a node acts as a bridge along the shortest paths between two other nodes. A node with high BC has great influence on what flows in the network and has more control over the network. It can represent the bottleneck in the network.

Eccentricity and CC of a node are the measures of centrality in the network, defined as the maximum distance from the node to all other nodes and the inverse of the average length of the shortest paths between the node and all other nodes, respectively. A node with lower eccentricity or higher CC is closer to the other nodes and more central in the network. Moreover, the maximum eccentricity is the diameter of a network; the minimum eccentricity is the radius of a network. The center of a network is the set of nodes of eccentricity equal to the radius.

EC assigns relative score to all nodes in the network based on the concept that connections to high-scoring nodes contribute more to the score of the node in question than equal connections to low-scoring nodes. Clustering coefficient of a node is the proportion of the edges to all the possible edges within its neighbors, quantifying the closeness among its neighbors, and evaluating how small its neighbors’ world is. A node with higher clustering coefficient has its neighbors closer to one another, and the world of its neighbors is smaller.

Global topological measurements of networks include average degree (<*k*>), mean shortest path length (mspl), diameter (D), and average clustering coefficient (acc). The clustering coefficient is a measure of the local interconnectedness of the graph, whereas the shortest path length is an indicator of its overall connectedness. A graph is considered small-world if it has a low mspl and a high acc^49–51^. According to Watts and Strogatz, small-world networks are a class of networks that are “highly clustered, like regular lattices, yet have small characteristic path lengths, like random graphs”.

### The retrieval of the backbone from the PPI network

We considered the protein nodes with high degree (*k*) or BC as the hubs or bottlenecks. They were key to the PPI network and constituted the backbone of the network^27^. Given the PPI network, we retrieved the protein nodes with top 10% highest degree (*k*) or BC, and defined the graph of these proteins as the backbone. With these proteins as input, we used STRING^45^ again to construct the 2^nd^ extended PPI network for further comprehensive analysis.

## Results

### The giant component of the PPI network

The giant component (Fig. 1) of the PPI network generated by STRING^45^ consisted of 111 nodes (Table S2) and 553 edges. The results of the topological analyses of each node were list in Table S3, including degree, BC, eccentricity, CC, EC, clustering coefficient, etc. The number of edges is larger than the expected for random network of the same size significantly (*p*-value ≤ 10^−16^); the nodes were more connected than randomly. It suggested that the PPI network could be considered as a relatively small world in comparison with random graph, and the proteins might be biologically relevant. The similar findings were also revealed in the results of the global topological measurements including average degree, mspl, diameter D, and average clustering coefficient listed in Table 1. In addition, the giant component of the PPI network with co-expression interactions (Fig. S1; Tables S4, S5) demonstrated the similar results, too.

**Table 1:** Global topological measurements of four PPI networks.

Symbol	Description	Giant component	Giant component (co-expression)	2^nd^ extended network	2^nd^ extended network (co-expression)
N	number of nodes	111	111	116	116
<*k*>	average degree	9.96 ± 7.32	9.24 ± 5.94	10.86 ± 9.14	
D	diameter	7	8	6	7
mspl	mean shortest path length	3.31	3.62	2.56	2.71
acc	average clustering coefficient	0.672	0.707	0.647	0.7640

Note: The average degrees of 16 key proteins in the giant component are 21.31 ± 9.78 and 18.88 ± 7.80.

### The backbone in the PPI network

**Figure 2:**
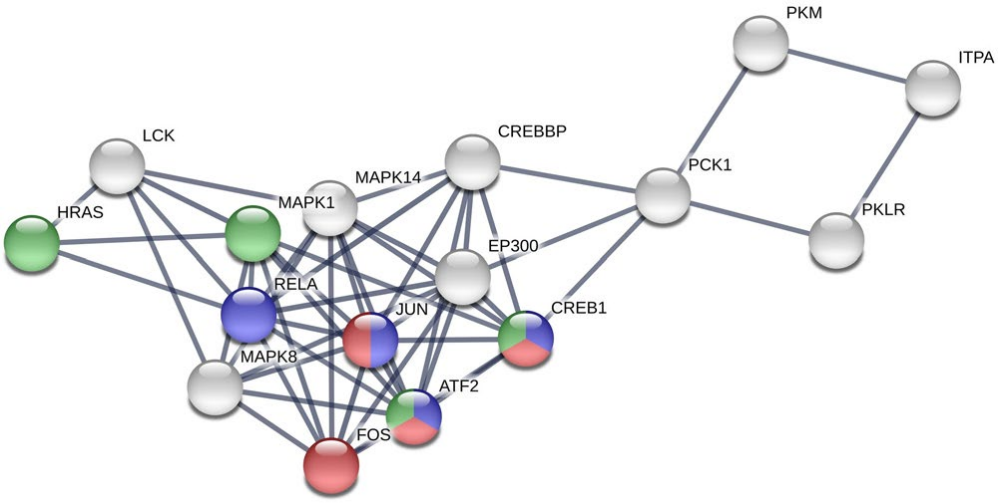
The backbone network.

The results of the topological analyses of each node in Table S3 showed that JUN was a hub (with the largest degree *k* = 43) and bottleneck (with the fourth highest BC = 0.1383) in the PPI network; PCK1 was only a bottleneck (with the highest BC = 0.35) but not a hub (with lower degree than average). We retrieved JUN, PCK1, and the other 14 proteins with top 10% largest degree (*k*) or highest BC and considered them as the hubs or bottlenecks and constituted the backbone of the giant component network (Fig. 2; Tables 2 and S6–S7). They were extensively connected with their neighbors in the network (the average degree: 21.31 ± 9.78) and had very much control over the network (the average BC: 0.11 ± 0.08). The backbone network consisted of 51 edges and 16 nodes. Among them, six proteins, JUN, MAPK14, CREEP, EP300, FOS, and RELA were both with top 10% largest degree (*k*) and highest BC, while PCK1 remained the most important bottleneck in the backbone.

**Table 2:** The proteins in the backbone network.

Label	Name	Description	Degree	Betweenness centrality
1	**JUN**	Jun proto-oncogene	43	0.138335165
2	**MAPK14**	Mitogen-activated protein kinase 14	32	0.083166142
3	**FOS**	FBJ murine osteosarcoma viral oncogene homolog	26	0.059760577
4	LCK	Lymphocyte-specific protein tyrosine kinase	26	0.058523406
5	**RELA**	V-rel reticuloendotheliosis viral oncogene homolog A (avian)	25	0.069572515
6	**MAPK1**	Mitogen-activated protein kinase 1	25	0.046624799
7	**CREBBP**	CREB binding protein	24	0.163843625
8	**ATF2**	Activating transcription factor 2	24	0.022118131
9	**EP300**	E1A binding protein p300	23	0.129821489
10	**MAPK8**	Mitogen-activated protein kinase 8	22	0.044774657
11	HRAS	v-Ha-ras Harvey rat sarcoma viral oncogene homolog	22	0.027715087
12	CREB1	cAMP responsive element binding protein 1	17	0.128192989
13	**ITPA**	Inosine triphosphatase (nucleoside triphosphate pyrophosphatase)	13	0.181820962
14	**PCK1**	Phosphoenolpyruvate carboxykinase 1 (soluble)	7	0.354489853
15	**PKLR**	Pyruvate kinase, liver and RBC	6	0.135446205
16	**PKM**	Pyruvate kinase, muscle	6	0.135446205
Average			21.31 ± 9.78	0.112282 ± 0.082026

Note: The bold ones are both in the backbone networks with and without co-expression setting in the STRING.

We also retrieved the backbone of the PPI network with co-expression interactions (Fig. S2 and Table S8). Comparing the two backbones with and without co-expression interactions, we discovered 13 proteins in common, and the 13-protein-subnetworks in the two backbones had the same structure (Fig. S3). The 13-protein-subnetworks contained most nodes and edges of the backbones. As a result, the main part of the backbones was robust, no matter with or without co-expression interactions derived from gene expression data. It suggested that only the rest part of the backbones could be influenced by the perturbations of gene expression through co-expression interactions.

### The 2^nd^ extended PPI network

**Figure 3:**
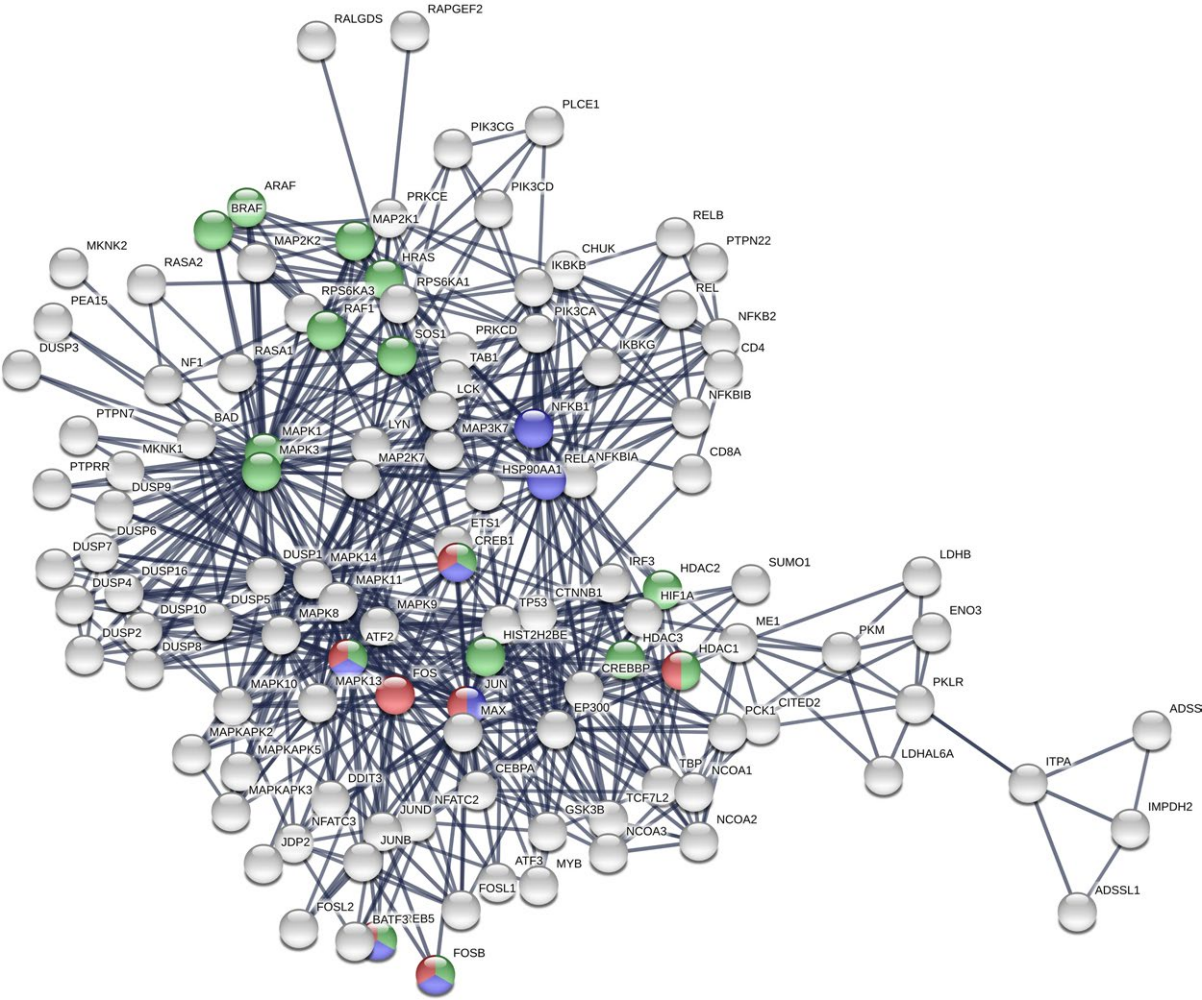
The 2^nd^ extended PPI network. *The red ones are nodes in amphetamine addiction pathway. ^+^The blue ones are nodes in cocaine addiction pathway. ^#^The green ones are nodes in alcoholism pathway.

With use of the backbone nodes as input, we implemented STRING again to construct the 2^nd^ extended PPI network associated with HUD. The global topological characteristics of four PPI networks in this study were listed in Table 1. The 2^nd^ extended PPI network without co-expression interactions demonstrated 16 nodes for the KEGG pathways^52^ of alcoholism (ARAF, ATF2, BRAF, CREB1, CREB5, FOSB, HDAC1, HDAC2, HDAC3, HIST2H2BE, HRAS, MAP2K1, MAPK1, MAPK3, RAF1, and SOS1), 7 nodes of amphetamine addiction (ATF2, CREB1, CREB5, FOS, FOSB, HDAC1, and JUN), and 7 nodes of cocaine addiction (ATF2, CREB1, CREB5, FOSB, JUN, NFKB1, and RELA) in Table 3. The nodes of the 2^nd^ extended PPI network involved in the KEGG pathways of amphetamine addiction, cocaine addiction, and alcoholism were colored in red, blue, and green, respectively, in Fig. 3. Moreover, the 2^nd^ extended PPI network with co-expression interactions was Fig. S4 which demonstrated 10 nodes for the KEGG pathways of alcoholism, 5 nodes of amphetamine addiction, and 6 nodes of cocaine addiction in Table S9. In addition, the three KEGG pathways^52^ were shown in Figs S5–S7.

**Table 3:** The proteins in the 2^nd^ extended PPI network involved in the KEGG pathways of alcoholism, amphetamine addiction, and cocaine addition.

KEGG pathway	Name	Description
Alcoholism	ARAF	V-raf murine sarcoma 3611 viral oncogene homolog
Alcoholism	ATF2	Activating transcription factor 2
Alcoholism	BRAF	V-raf murine sarcoma viral oncogene homolog B1
Alcoholism	CREB1	cAMP responsive element binding protein 1
Alcoholism	CREB5	cAMP responsive element binding protein 5
Alcoholism	FOSB	FBJ murine osteosarcoma viral oncogene homolog B
Alcoholism	HDAC1	Histone deacetylase 1
Alcoholism	HDAC2	Histone deacetylase 2
Alcoholism	HDAC3	Histone deacetylase 3
Alcoholism	HIST2H2BE	Histone cluster 2, H2be
Alcoholism	HRAS	v-Ha-ras Harvey rat sarcoma viral oncogene homolog
Alcoholism	MAPK1	Mitogen-activated protein kinase 1
Alcoholism	MAP2K1	Mitogen-activated protein kinase kinase 1
Alcoholism	MAPK3	Mitogen-activated protein kinase 3
Alcoholism	RAF1	V-raf-1 murine leukemia viral oncogene homolog 1
Alcoholism	SOS1	Son of sevenless homolog 1 (Drosophila)
Amphetamine	ATF2	Activating transcription factor 2
Amphetamine	CREB1	cAMP responsive element binding protein 1
Amphetamine	CREB5	cAMP responsive element binding protein 5
Amphetamine	FOS	FBJ murine osteosarcoma viral oncogene homolog
Amphetamine	FOSB	FBJ murine osteosarcoma viral oncogene homolog B
Amphetamine	JUN	Jun proto-oncogene
Amphetamine	HDAC1	Histone deacetylase 1
Cocaine	ATF2	Activating transcription factor 2
Cocaine	CREB1	cAMP responsive element binding protein 1
Cocaine	CREB5	cAMP responsive element binding protein 5
Cocaine	FOSB	FBJ murine osteosarcoma viral oncogene homolog B
Cocaine	JUN	Jun proto-oncogene
Cocaine	NFKB1	Nuclear factor of kappa light polypeptide gene enhancer in B-cells 1
Cocaine	RELA	V-rel reticuloendotheliosis viral oncogene homolog A (avian)

## Discussion

Several studies have been conducted on HUD and several related susceptibility genes have been reported; however, the potential mechanism underlying HUD development remains unclear^53,54^. The proteins encoded by susceptibility genes may determine an individual’s susceptibility to HUD through their encoded PPIs. Here, we studied the potential key proteins through topological analysis^18,55,56^. We used degree (*k*) and BC, the 2 measures widely used in network theory, as the main parameters for evaluating the nodes in the PPI network^27^.

This is the first study to investigate the PPI network of HUD and explore the contributions of the proteins encoded by the susceptibility genes associated with HUD. Initially, a total of 111 proteins were included in our giant component network. By considering the important topological measures (degree and BC) in a large network, we selected 16 proteins to construct the backbone network: 11 with large degree, 11 with high BC, and 4 with both large degree and high BC. In the PPI network, there were 16 proteins involved in the alcoholism pathway, 7 proteins involved in the amphetamine addiction pathway, and 7 proteins involved in the cocaine addiction pathway in the KEGG. Nevertheless, although both morphine and heroin are synthesized from opium, proteins involved in the KEGG pathway of morphine addiction were absent in the network. According to gateway drug theory, alcohol and cannabis are frequently abused before illicit drugs such as cocaine and heroin. Cannabis use is not popular in Taiwan; therefore, the 16 proteins involved in alcoholism might be important for the further development of HUD. In Taiwan, the frequency of cocaine use is low; however, the frequency of amphetamine use, a cocaine-like stimulant, is high in clients with HUD^57^. It might be due to most individuals with HUD abusing other substance such as amphetamine^58,59^.

Seven nodes including JUN, FOS, RELA, MAPK1, ATF2, HRAS, and CREB1 in the backbone are the recorded genes of alcoholism, amphetamine addiction, or cocaine addiction in the KEGG pathways. They are the identified key proteins in substance diseases involved in HUD development revealed in this study.

The most important one among them is JUN, one of the biosignatures for detecting HUD in men^43^ and a seed protein in the initial PPI network. JUN (with the largest degree and the fourth highest BC) is central to the PPI network, the backbone network and the 2^nd^ extended PPI network. It has been implicated in cancer-related studies^60^ as well as studies on psychological disorders such as Alzheimer disease and schizophrenia^61,62^. JUN has been reported to be involved in amphetamine and cocaine addiction and their respective KEGG pathways^63,64^.

FOS, RELA, and HRAS in the backbone are known to be in the KEGG pathway associated with substance diseases. RELA, or v-rel avian reticuloendotheliosis viral oncogene homolog A, is also named as p65 or NF-κB (nuclear factor kappa-light-chain-enhancer of activated B cells) is a protein complex that controls transcription of DNA, cytokine production and cell survival and was found to be correlated with cancers and Alzheimer’s disease^65–68^. RELA (NF-κB) is known as an induced transcriptional targets of ΔFosB associated with addiction to a stimulus such as cocaine and the effect of reward system^69–72^. HRAS is a small G protein in the Ras subfamily associated small GTPases, and familial alcohol dependence was associated with hypomethylation of CpG sites in the HRAS promoter region^73^. FOS is a 380 amino acid protein with a basic leucine zipper region for dimerisation and DNA-binding and a transactivation domain at C-terminus and FOS is unable to make FOS-FOS homodimers. JUN–FOS heterodimers are more stable and have stronger DNA-binding activity than JUN–JUN homodimers. FOS is known to have interaction in an animal study^74^. Moreover, ATF2, CREB1, CREB5, and FOSB in all three substance diseases (alcoholism, amphetamine, and cocaine addiction pathways). All of them except FOSB are selected in to the backbone network. CREB1 and CREB5 belong to CREB (cAMP response element-binding protein) family and are correlated with substance diseases^75–80^. FOSB belongs to FOS gene family including FOS. The FOS family play a role in the development and maintenance of drug addictions^70,71^. ATF2 interacting with JUN, CREB family, and other proteins in the backbone network would be discussed latter^81^.

Some other nodes in the backbone network are worth to study although they are not recorded in the alcoholism, amphetamine addiction, and cocaine addiction pathways. MAPK14, is not *recognized* in substance diseases in these KEGG pathways. However, MAPK14, also called p38-α, is an enzyme in humans encoded by the MAPK14 gene and a member of the MAP kinase family. The substrates of this kinase also include transcription regulator ATF2 on stress-activated protein kinases^82^. ATF2 is in the backbone network and is involved in all three substance diseases (alcoholism, amphetamine addiction, and cocaine addiction) in the KEGG pathways. PCK1, an enzyme in humans encoded by the PCK1 gene, is an important control point for the regulation of gluconeogenesis. PCK1 and MAPK14 were not found to be involved in any substance diseases previously but they were noticed in the study of schizophrenia^83^. MAPK14 has the 2^nd^ large degree and 9^th^ high BC and PCK1 has the highest BC in our giant component network. The two nodes provide us the new cues for further study in HUD and other substance diseases.

In addition, changes in miRNA expression levels are linked to neurodegeneration^84^ with mounting of evidence supporting the dysregulation of miRNA expression in psychiatric and neurological disorders^35–37,85–87^. MiRNAs might play important functions in moderating the central stress response within different brain regions via the regulation of genes^35^. As a result, miRNAs can not only serve as biomarkers of addition, but also as promising therapies for the prevention or treatment of neurodevelopmental and neuropathological disorders^37^. Based on proteins in Table 2, we found that some miRNAs target FOS, JUN, MAPK1, MAPK14, and RELA (Table S10) in the study of the brains genomic response to environmental stress^35^, and some of them involve in addiction such as cocaine, alcohol^37,85^. We searched for miRNA-substance-use-disorder associations from HMDD v3.0 database^42^, and there were few information related to substance use disorders (Table S11). In addition, researches of the miRNA-HUD associations are few^38^, therefore, it still remains much unknown. The recent advances of specific miRNAs have emerged as key regulator leading to addiction, and further studies may be central for developing novel therapeutic approaches^85^. Implementing predictive computational models might be potential to discovery miRNA biomarkers for HUD in the future^39–42,88^.

A limitation of this study is the lack of proteins strongly correlated with morphine addiction or HUD in the backbone network. This may be due to early-life exposure to alcohol or amphetamine having a greater impact on persons with HUD than later-life heroin use. Another limitation of this study is that we used peripheral blood as the sample, rather than brain tissue from areas such as nucleus accumbens^89^, based on a previous human study^90^.

## Conclusion

Our finding suggests that HUD develops through an integrated PPI network with JUN (the largest degree and the 4^th^ highest BC) at its center. JUN is also involved in the development of amphetamine and cocaine addiction. However, the role of JUN in HUD requires further clarification. FOS, RELA, ATF2, and HRAS in the backbone network are also recorded of one of three substance diseases in KEGG pathways. (Alcoholism, amphetamine addiction, cocaine addiction) ATF2, CREB1, CREB5, and FOSB in all three substance disease KEGG pathways are suspected be related with CREB and FOS family, and JUN. Furthermore, MAPK14 (the second largest degree and the 9^th^ high BC) and PCK1 (the highest BC) have a major role in the PPI network. The present study marks the beginning of the formulation of the PPI network of HUD, with JUN and other two key proteins MAPK14 and PCK1.

## Supplementary information

Supplementary

Table S3

Table S5

Table S11
