# Supplementary material for: Construction and Analysis of Protein-Protein Interaction Network of Heroin Use Disorder
Source: Sci Rep. 2019 Mar 21;9:4980. doi: 10.1038/s41598-019-41552-z (PMC6428805; doi:10.1038/s41598-019-41552-z)
Supplement: Supplementary file 1 — Supplementary [file 41598_2019_41552_MOESM1_ESM.pdf]

Supplementary Information

Construction and Analysis of Protein-Protein Interaction Network of Heroin Use Disorder

Shaw-Ji Chen[1,2], Ding-Lieh Liao[3], Chia-Hsiang Chen[4],

Tse-Yi Wang[5], Kuang-Chi Chen[5 *]

[1] Department of Medicine, Mackay Medical College, New Taipei City, Taiwan,

[2] Department of Psychiatry, Mackay Memorial Hospital, Taitung Branch, Taiwan

[3] Bali Psychiatric Center, Department of Health, Executive Yuan, New Taipei, Taiwan

[4] Department of Psychiatry, Chang Gung Memorial Hospital at Linkou and Chang Gung University School of Medicine, Taoyuan, Taiwan

[5]Department of Medical Informatics, Tzu Chi University, Hualien, Taiwan

Please address correspondence to:
Kuang-Chi Chen Ph.D.
Dept. of Medical Informatics, Tzu Chi University, Hualien, Taiwan, R.O.C.
Phone: 886-3- 8565301 # 2390
Fax: 886-3- 8579409
E-mail: chichen6@mail.tcu.edu.tw

Legend of Tables

Table S1. The seed proteins associated with HUD

Table S2. The proteins in PPI network extracted from STRING database associated with HUD

Table S3. The topological measurements of PPI network

Table S4. The proteins in the PPI network (with co-expression) extracted from STRING database associated with HUD

Table S5. The topological measurements of PPI network (with co-expression)

Table S6. Top 25 nodes with high degree and their respective BC and CC

Table S7. Top 25 nodes with high BC and their respective *k* and CC

Table S8. The proteins in the backbone network (with co-expression)

Table S9. The proteins in the 2nd extended PPI network (with co-expression) involved in the KEGG pathways of alcoholism, amphetamine addiction, and cocaine addition

Table S10. The miRNA targeting genes by stress-induced

Table S11. The miRNA-disease associations in HMDD v3.0

Legend of Figures

Figure S1. The PPI network (with co-expression)

Figure S2. The backbone network (with co-expression)

Figure S3. The 13-protein-subnetwork of the two backbones

Figure S4. The 2nd extended PPI network (with co-expression)

*The red ones are nodes in amphetamine addiction pathway

+The blue ones are nodes in cocaine addiction pathway

#The green ones are nodes in alcoholism pathway

Figure S5. The KEGG pathways of alcoholism

Figure S6. The KEGG pathways of amphetamine addiction

Figure S7. The KEGG pathways of cocaine addiction

Table S1. The seed proteins associated with HUD

Label	Name	Description
1	AUTS2	autism susceptibility candidate 2
2	CD74	CD74 molecule, major histocompatibility complex, class II invariant chain
3	CEBPB	CCAAT/enhancer binding protein (C/EBP), beta
4	CEBPG	CCAAT/enhancer binding protein (C/EBP), gamma
5	ENO2	enolase 2 (gamma, neuronal)
6	HAT1	histone acetyltransferase 1
7	IMPDH2	IMP (inosine 5'-monophosphate) dehydrogenase 2
8	JUN	jun proto-oncogene
9	MBD1	methyl-CpG binding domain protein 1
10	PDK1	pyruvate dehydrogenase kinase, isozyme 1
11	PRKCB	protein kinase C, beta
12	RASA1	RAS p21 protein activator
13	RGS3	regulator of G-protein signaling 3

Table S2. The proteins in the PPI network extracted from STRING database associated with HUD

Label	Names	Description
1	ARHGAP35	Rho GTPase activating protein 35
2	ATF1	Activating transcription factor 1
3	ATF2	Activating transcription factor 2
4	ATF3	Activating transcription factor 3
5	ATIC	5-aminoimidazole-4-carboxamide ribonucleotide formyltransferase/IMP cyclohydrolase
6	AURKB	Aurora kinase B
7	BATF3	Basic leucine zipper transcription factor, ATF-like 3; AP-1 family transcription factor that controls the
8	BPGM	2,3-bisphosphoglycerate mutase
9	CD74	CD74 molecule, major histocompatibility complex, class II invariant chain
10	CEBPA	CCAAT/enhancer binding protein (C/EBP), alpha
11	CEBPB	CCAAT/enhancer binding protein (C/EBP), betaplays essential and redundant functions with CE [...]
12	CEBPD	CCAAT/enhancer binding protein (C/EBP), delta heterodimer with CEBPB (PubMed:1741402)
13	CEBPG	CCAAT/enhancer binding protein (C/EBP), gamma promoter
14	CHUK	Conserved helix-loop-helix ubiquitous kinase
15	COPS5	COP9 constitutive photomorphogenic homolog subunit 5 (Arabidopsis)
16	CREB1	cAMP responsive element binding protein 1
17	CREBBP	CREB binding protein
18	CSK	C-src tyrosine kinase
19	CTSL1	Cathepsin L1; Important for the overall degradation of proteins in lysosomes
20	DLAT	Dihydrolipoamide S-acetyltransferase
21	DOK1	Docking protein 1, 62kDa (downstream of tyrosine kinase 1)
22	DOK2	Docking protein 2, 56kDa
23	DUSP1	Dual specificity phosphatase 1
24	DVL2	Dishevelled, dsh homolog 2
25	EGFR	Epidermal growth factor receptor
26	EGR1	Early growth response 1
27	ENO2	Enolase 2 (gamma, neuronal)
28	ENO3	Enolase 3 (beta, muscle)
29	ENSG00000250741	NT5C1B-RDH14 readthroug
30	EP300	E1A binding protein p300
31	EPHB2	EPH receptor B2
32	EPHB3	EPH receptor B3
33	ESR1	Estrogen receptor 1

34	ETS1	V-ets erythroblastosis virus E26 oncogene homolog 1 (avian);
35	ETS2	V-ets erythroblastosis virus E26 oncogene homolog 2 (avian)
36	FOS	FBJ murine osteosarcoma viral oncogene homolog
37	FOSB	FBJ murine osteosarcoma viral oncogene homolog B
38	FOSL1	FOS-like antigen 1
39	FOSL2	FOS-like antigen 2
40	GATA2	GATA binding protein 2
41	GMPR	Guanosine monophosphate reductase
42	GMPR2	Guanosine monophosphate reductase 2
43	GMPS	Guanine monphosphate synthetase;
44	GNA11	Guanine nucleotide binding protein (G protein), alpha 11 (Gq class)
45	GNA14	Guanine nucleotide binding protein (G protein), alpha 14
46	GNA15	Guanine nucleotide binding protein (G protein), alpha 15 (Gq class)
47	GNAQ	Guanine nucleotide binding protein (G protein), q polypeptide
48	GNB2L1	Guanine nucleotide binding protein (G protein), beta polypeptide 2-like 1
49	GSK3B	Glycogen synthase kinase 3 beta
50	GTF2B	General transcription factor IIB
51	HAT1	Histone acetyltransferase 1
52	HCK	Hemopoietic cell kinase
53	HLA-DMB	Major histocompatibility complex, class II, DM beta
54	HLA-DPA1	Major histocompatibility complex, class II, DP alpha 1
55	HLA-DRA	Major histocompatibility complex, class II, DR alpha
56	HLA-DRB1	Major histocompatibility complex, class II, DR beta 1
57	HLA-DRB5	Major histocompatibility complex, class II, DR beta 5
58	HPRT1	Hypoxanthine phosphoribosyltransferase 1
59	HRAS	v-Ha-ras Harvey rat sarcoma viral oncogene homolog
60	IKBKB	Inhibitor of kappa light polypeptide gene enhancer in B-cells, kinase beta
61	IMPDH1	IMP (inosine 5'-monophosphate) dehydrogenase 1
62	IMPDH2	IMP (inosine 5'-monophosphate) dehydrogenase 2
63	ITPA	Inosine triphosphatase (nucleoside triphosphate pyrophosphatase)
64	JDP2	Jun dimerization protein 2
65	JUN	Jun proto-oncogene
66	KAT2B	K(lysine) acetyltransferase 2
67	LCK	Lymphocyte-specific protein tyrosine kinase
68	MAFB	V-maf musculoaponeurotic fibrosarcoma oncogene homolog B (avian)
69	MAPK1	Mitogen-activated protein kinase 1

70	MAPK10	Mitogen-activated protein kinase 10
71	MAPK11	Mitogen-activated protein kinase 11
72	MAPK14	Mitogen-activated protein kinase 14
73	MAPK3	Mitogen-activated protein kinase 3
74	MAPK8	Mitogen-activated protein kinase 8
75	MAPK9	Mitogen-activated protein kinase 9
76	MYB	V-myb myeloblastosis viral oncogene homolog (avian)
77	NFATC2	Nuclear factor of activated T-cells, cytoplasmic, calcineurin-dependent 2
78	NFE2L2	Nuclear factor (erythroid-derived 2)-like 2
79	NR3C1	Nuclear receptor subfamily 3, group C, member 1 (glucocorticoid receptor)
80	NT5C1A	5'-nucleotidase, cytosolic IA
81	NT5C2	5'-nucleotidase, cytosolic II
82	NT5E	5'-nucleotidase, ecto (CD73)
83	PCK1	Phosphoenolpyruvate carboxykinase 1 (soluble)
84	PDGFRB	Platelet-derived growth factor receptor, beta polypeptide
85	PDHA1	Pyruvate dehydrogenase (lipoamide) alpha 1
86	PDHB	Pyruvate dehydrogenase (lipoamide) beta
87	PDHX	Pyruvate dehydrogenase complex, component X
88	PDK1	Pyruvate dehydrogenase kinase, isozyme 1
89	PDPK1	3-phosphoinositide dependent protein kinase-1
90	PGAM1	Phosphoglycerate mutase 1 (brain)
91	PGAM2	Phosphoglycerate mutase 2 (muscle)
92	PGAM4	Phosphoglycerate mutase family member 4
93	PKLR	Pyruvate kinase, liver and RBC;
94	PKM	Pyruvate kinase, muscle
95	PRKCB	Protein kinase C, beta
96	PRKCD	Protein kinase C, delta
97	RASA1	RAS p21 protein activator (GTPase activating protein) 1
98	RBBP4	Retinoblastoma binding protein 4
99	RBBP7	Retinoblastoma binding protein 7
100	RELA	V-rel reticuloendotheliosis viral oncogene homolog A (avian)
101	RGS3	Regulator of G-protein signaling 3
102	RPS6KA1	Ribosomal protein S6 kinase, 90kDa, polypeptide 1
103	SHC1	SHC (Src homology 2 domain containing) transforming protein 1
104	SMAD3	SMAD family member 3
105	SMARCA4	SWI/SNF related, matrix associated, actin dependent regulator of chromatin, subfamily a, member 4
106	SMARCB1	SWI/SNF related, matrix associated, actin dependent regulator of

		chromatin, subfamily b, member 1
107	SMARCD1	SWI/SNF related, matrix associated, actin dependent regulator of chromatin, subfamily d, member 1
108	SP1	Sp1 transcription factor
109	SRC	V-src sarcoma (Schmidt-Ruppin A-2) viral oncogene homolog (avian)
110	STAT4	Signal transducer and activator of transcription 4
111	YES1	V-yes-1 Yamaguchi sarcoma viral oncogene homolog 1

seed	AUTS2	Autism susceptibility candidate 2

seed	MBD1	methyl-CpG binding domain protein 1

Table S4. The proteins in the PPI network (with co-expression) extracted from STRING database associated with HUD

Label	Names	Description
1	ARHGAP35	Rho GTPase activating protein 35
2	ATF2	Activating transcription factor 2
3	ATF3	Activating transcription factor 3
4	ATIC	5-aminoimidazole-4-carboxamide ribonucleotide formyltransferase/IMP cyclohydrolase
5	BATF3	Basic leucine zipper transcription factor, ATF-like 3
6	BPGM	2,3-bisphosphoglycerate mutase
7	CAD	Carbamoyl-phosphate synthetase 2, aspartate transcarbamylase, and dihydroorotase
8	CD74	CD74 molecule, major histocompatibility complex, class II invariant chain
9	CEBPA	CCAAT/enhancer binding protein (C/EBP), alpha
10	CEBPB	CCAAT/enhancer binding protein (C/EBP), betaplays essential and redundant functions with CE [...]
11	CEBPD	CCAAT/enhancer binding protein (C/EBP), delta heterodimer with CEBPB (PubMed:1741402)
12	CEBPG	CCAAT/enhancer binding protein (C/EBP), gamma promoter
13	CHUK	Conserved helix-loop-helix ubiquitous kinase
14	COPS5	COP9 constitutive photomorphogenic homolog subunit 5 (Arabidopsis)
15	CPS1	Carbamoyl-phosphate synthase 1
16	CREB1	cAMP responsive element binding protein 1
17	CREBBP	CREB binding protein
18	CTPS1	CTP synthase 1
19	CTPS2	CTP synthase 2
20	CTSL1	Cathepsin L1; Important for the overall degradation of proteins in lysosomes
21	DLAT	Dihydrolipoamide S-acetyltransferase
22	DOK1	Docking protein 1, 62kDa (downstream of tyrosine kinase 1)
23	DOK2	Docking protein 2, 56kDa
24	DUSP1	Dual specificity phosphatase 1
25	DVL2	Dishevelled, dsh homolog 2
26	EGR1	Early growth response 1
27	ENO2	Enolase 2 (gamma, neuronal)
28	ENO3	Enolase 3 (beta, muscle)
29	ENSG00000 250741	NT5C1B-RDH14 readthroug
30	EP300	E1A binding protein p300
31	EPHB2	EPH receptor B2
32	EPHB3	EPH receptor B3

33	ESR1	Estrogen receptor 1
34	ETS1	V-ets erythroblastosis virus E26 oncogene homolog 1 (avian);
35	ETS2	V-ets erythroblastosis virus E26 oncogene homolog 2 (avian)
36	FOS	FBJ murine osteosarcoma viral oncogene homolog
37	FOSB	FBJ murine osteosarcoma viral oncogene homolog B
38	FOSL1	FOS-like antigen 1
39	FOSL2	FOS-like antigen 2
40	GAPDH	Glyceraldehyde-3-phosphate dehydrogenase
41	GAPDHS	Glyceraldehyde-3-phosphate dehydrogenase, spermatogenic
42	GMPR	Guanosine monophosphate reductase
43	GMPR2	Guanosine monophosphate reductase 2
44	GMPS	Guanine monphosphate synthetase;
45	GNA11	Guanine nucleotide binding protein (G protein), alpha 11 (Gq class)
46	GNA14	Guanine nucleotide binding protein (G protein), alpha 14
47	GNA15	Guanine nucleotide binding protein (G protein), alpha 15 (Gq class)
48	GNAQ	Guanine nucleotide binding protein (G protein), q polypeptide
49	GPI	Glucose-6-phosphate isomerase
50	GSK3B	Glycogen synthase kinase 3 beta
51	HAT1	Histone acetyltransferase 1
52	HLA-DMA	Hemopoietic cell kinase
53	HLA-DMB	Major histocompatibility complex, class II, DM beta
54	HLA-DPA1	Major histocompatibility complex, class II, DP alpha 1
55	HLA-DPB1	Major histocompatibility complex, class II, DR alpha
56	HLA-DQA2	Major histocompatibility complex, class II, DR beta 1
57	HLA-DRA	Major histocompatibility complex, class II, DR beta 5
58	HLA-DRB1	Major histocompatibility complex, class II, DR beta 1
59	HLA-DRB5	Major histocompatibility complex, class II, DR beta 5
60	HPRT1	Hypoxanthine phosphoribosyltransferase 1
61	HRAS	v-Ha-ras Harvey rat sarcoma viral oncogene homolog
62	IKBKB	Inhibitor of kappa light polypeptide gene enhancer in B-cells, kinase beta
63	IMPDH1	IMP (inosine 5'-monophosphate) dehydrogenase 1
64	IMPDH2	IMP (inosine 5'-monophosphate) dehydrogenase 2
65	ITPA	Inosine triphosphatase (nucleoside triphosphate pyrophosphatase)
66	JDP2	Jun dimerization protein 2
67	JUN	Jun proto-oncogene L
68	JUNB	V-maf musculoaponeurotic fibrosarcoma oncogene homolog B (avian)
69	KAT2B	K(lysine) acetyltransferase 2

70	MAFB	V-maf musculoaponeurotic fibrosarcoma oncogene homolog B (avian)
71	MAPK1	Mitogen-activated protein kinase 10
72	MAPK10	Mitogen-activated protein kinase 11
73	MAPK11	Mitogen-activated protein kinase 14
74	MAPK14	Mitogen-activated protein kinase 3
75	MAPK8	Mitogen-activated protein kinase 8
76	MAPK9	Mitogen-activated protein kinase 9
77	MYB	V-myb myeloblastosis viral oncogene homolog (avian)
78	NFATC2	Nuclear factor (erythroid-derived 2)-like 2
79	NR3C1	Nuclear receptor subfamily 3, group C, member 1 (glucocorticoid receptor)
80	NT5C1A	5'-nucleotidase, cytosolic IA
81	NT5C2	5'-nucleotidase, cytosolic II
82	NT5E	5'-nucleotidase, ecto (CD73)
83	PCK1	Phosphoenolpyruvate carboxykinase 1 (soluble)
84	PDHA1	Pyruvate dehydrogenase (lipoamide) alpha 1
85	PDHB	Pyruvate dehydrogenase (lipoamide) beta
86	PDHX	Pyruvate dehydrogenase complex, component X
87	PDK1	Pyruvate dehydrogenase kinase, isozyme 1
88	PDPK1	3-phosphoinositide dependent protein kinase-1
89	PGAM1	Phosphoglycerate mutase 1 (brain)
90	PGAM2	Phosphoglycerate mutase 2 (muscle)
91	PGAM4	Phosphoglycerate mutase family member 4
92	PGK1	Phosphoglycerate kinase 1
93	PGK2	Phosphoglycerate kinase 2
94	PKLR	Pyruvate kinase, liver and RBC
95	PKM	Pyruvate kinase, muscle
96	PNMAL2	Paraneoplastic Ma antigen family-like 2
97	PRKCB	Protein kinase C, beta
98	PRKCD	Protein kinase C, delta
99	RASA1	RAS p21 protein activator (GTPase activating protein) 1
100	RBBP4	Retinoblastoma binding protein 4
101	RBBP7	Retinoblastoma binding protein 7
102	RELA	V-rel reticuloendotheliosis viral oncogene homolog A (avian)
103	RGS3	Regulator of G-protein signaling 3
104	SMARCA4	SWI/SNF related, matrix associated, actin dependent regulator of chromatin, subfamily a, member 4
105	SMARCB1	SWI/SNF related, matrix associated, actin dependent regulator of

		chromatin, subfamily b, member 1
106	SMARCD1	SWI/SNF related, matrix associated, actin dependent regulator of chromatin, subfamily d, member 1
107	SP1	Sp1 transcription factor
108	SRC	V-src sarcoma (Schmidt-Ruppin A-2) viral oncogene homolog (avian)
109	STAT4	Signal transducer and activator of transcription 4
110	TPI1	Triosephosphate isomerase 1
111	YES1	V-yes-1 Yamaguchi sarcoma viral oncogene homolog 1

seed	AUTS2	Autism susceptibility candidate 2
seed	MBD1	methyl-CpG binding domain protein 1

Table S6. Top 25 nodes with high degree and their respective BC and CC

Label	Name	Degree	Betweenness centrality	Closeness centrality
1*	**JUN**	**43**	**0.138335165**	**0.436507937**
2	**MAPK14**	**32**	**0.083166142**	**0.433070866**
3	FOS	26	0.059760577	0.415094340
4	LCK	26	0.058523406	0.371621622
5	**RELA**	**25**	**0.069572515**	**0.421455939**
6	MAPK1	25	0.046624799	0.423076923
7	**CREBBP**	**24**	**0.163843625**	**0.452674897**
8	ATF2	24	0.022118131	0.402930403
9	EP300	23	0.129821489	0.443548387
10	MAPK8	22	0.044774657	0.418250951
11	HRAS	22	0.027715087	0.358306189
12*	RASA1	21	0.037173088	0.351437700
13	SRC	21	0.023429190	0.360655738
14	MAPK3	19	0.019268371	0.408921933
15	SHC1	18	0.030403520	0.390070922
16	CREB1	17	0.128192989	0.433070866
17	YES1	17	0.015116120	0.350318471
18	MAPK11	17	0.014441917	0.400000000
19	SP1	17	0.007943959	0.392857143
20	CSK	16	0.056993119	0.385964912
21	ESR1	16	0.023134181	0.395683453
22*	CEBPB	15	0.023990476	0.384615385
23	SMAD3	15	0.012296350	0.384615385
24	NR3C1	14	0.002076446	0.381944444
25	ITPA	13	0.181820962	0.250000000

Note: The bold ones have both large degree and high BC

The asterisk ones are the seed proteins (3 nodes: JUN, RASA1, CEBPB)

Table S7. Top 25 nodes with high BC and their respective *k* and CC

Label	Name	Degree	Betweenness centrality	Closeness centrality
1	PCK1	7	0.354489853	0.384615385
2	ITPA	13	0.181820962	0.250000000
3	**CREBBP**	**24**	**0.163843625**	**0.452674897**
4*	**JUN**	**43**	**0.138335165**	**0.436507937**
5	PKLR	6	0.135446205	0.309859155
6	PKM	6	0.135446205	0.309859155
7	EP300	23	0.129821489	0.443548387
8	CREB1	17	0.128192989	0.433070866
9	**MAPK14**	**32**	**0.083166142**	**0.433070866**
10	**RELA**	**25**	**0.069572515**	**0.421455939**
11	FOS	26	0.059760577	0.415094340
12	LCK	26	0.058523406	0.371621622
13	CSK	16	0.056993119	0.385964912
14	MAPK1	25	0.046624799	0.423076923
15	MAPK8	22	0.044774657	0.418250951
16*	RASA1	21	0.037173088	0.351437700
17	HLA-DRB1	10	0.036399507	0.332326284
18*	ENO2	8	0.035223798	0.299727520
19	ENO3	8	0.035223798	0.299727520
20	SHC1	18	0.030403520	0.390070922
21	HRAS	22	0.027715087	0.358306189
22	PDHA1	6	0.026549903	0.241758242
23	PDHB	6	0.026549903	0.241758242
24*	CEBPB	15	0.023990476	0.384615385
25	SRC	21	0.023429190	0.360655738

Note: The bold ones have both large degree and high BC

The asterisk ones are the seed proteins (4 nodes: JUN, RASA1, ENO2, CEBPB)

Table S8. The proteins in the backbone network (with co-expression)

Label	Name	Description	Degree	Betweenness centrality
1	**JUN**	Jun proto-oncogene	38	.120966583
2	**MAPK14**	Mitogen-activated protein kinase 14	27	.059088263
3	**FOS**	FBJ murine osteosarcoma viral oncogene homolog	24	.194660537
4	**EP300**	E1A binding protein p300	23	.169481630
5	**RELA**	V-rel reticuloendotheliosis viral oncogene homolog A (avian)	22	.084060558
6	**ATF2**	Activating transcription factor 2	22	.025940031
7	**CREBBP**	CREB binding protein	22	.119716044
8	**MAPK8**	Mitogen-activated protein kinase 8	21	.049177394
9	**MAPK1**	Mitogen-activated protein kinase 1	18	.040309594
10	SP1	Sp1 transcription factor	17	.009311159
11	ESR1	Estrogen receptor 1	16	.136690750
12	**ITPA**	Inosine triphosphatase (nucleoside triphosphate pyrophosphatase)	15	.238968585
13	**PKM**	Pyruvate kinase, muscle	10	.163451120
14	**PKLR**	Pyruvate kinase, liver and RBC	10	.163451120
15	HLA-DRB1	Major histocompatibility complex, class II, DR beta 1	10	.151674014
16	**PCK1**	Phosphoenolpyruvate carboxykinase 1 (soluble)	7	.322721318
Average			18.88±7.80	0.128104±0.083565

Note: The bold ones are both in the backbone networks with and without co-expression setting in the STRING

Table S9. The proteins in the 2nd extended PPI network (with co-expression) involved in the KEGG pathways of alcoholism, amphetamine addiction, and cocaine addition

KEGG pathway	Name	Description
Alcoholism	ATF2	Activating transcription factor 2
Alcoholism	BRAF	V-raf murine sarcoma viral oncogene homolog B1
Alcoholism	CREB5	cAMP responsive element binding protein 5
Alcoholism	FOSB	cAMP responsive element binding protein 1
Alcoholism	HDAC2	Histone deacetylase 2
Alcoholism	HDAC6	Histone deacetylase 6
Alcoholism	MAP2K1	Mitogen-activated protein kinase kinase 1
Alcoholism	MAPK1	Mitogen-activated protein kinase 1
Alcoholism	MAPK3	Mitogen-activated protein kinase 3
Alcoholism	RAF1	V-raf-1 murine leukemia viral oncogene homolog 1
Amphetamine	ATF2	Activating transcription factor 2
Amphetamine	CREB5	cAMP responsive element binding protein 5
Amphetamine	FOS	FBJ murine osteosarcoma viral oncogene homolog
Amphetamine	FOSB	FBJ murine osteosarcoma viral oncogene homolog B
Amphetamine	JUN	Jun proto-oncogene
Cocaine	ATF2	Activating transcription factor 2
Cocaine	CREB5	cAMP responsive element binding protein 5
Cocaine	FOSB	FBJ murine osteosarcoma viral oncogene homolog B
Cocaine	JUN	Jun proto-oncogene
Cocaine	NFKB1	Nuclear factor of kappa light polypeptide gene enhancer in B-cells 1
Cocaine	RELA	V-rel reticuloendotheliosis viral oncogene homolog A (avian)

Table S10. The miRNA targeting genes by stress-induced

Gene Name	Description	Stress-induced differentially expressed miRNA
FOS	FBJ murine osteosarcoma viral oncogene homolog	miR-221, miR-29a
JUN	jun proto-oncogene	miR-15b, miR-30a
MAPK1	mitogen-activated protein kinase 1	miR-124, miR-132, miR-15b, miR-106b, miR-186, miR-19a, miR-204, miR-26a, miR-410, miR-32
MAPK14	mitogen-activated protein kinase 14	miR-124, miR-351, miR-19a, miR-22
RELA	v-rel avian reticuloendotheliosis viral oncogene homolog A	miR-124

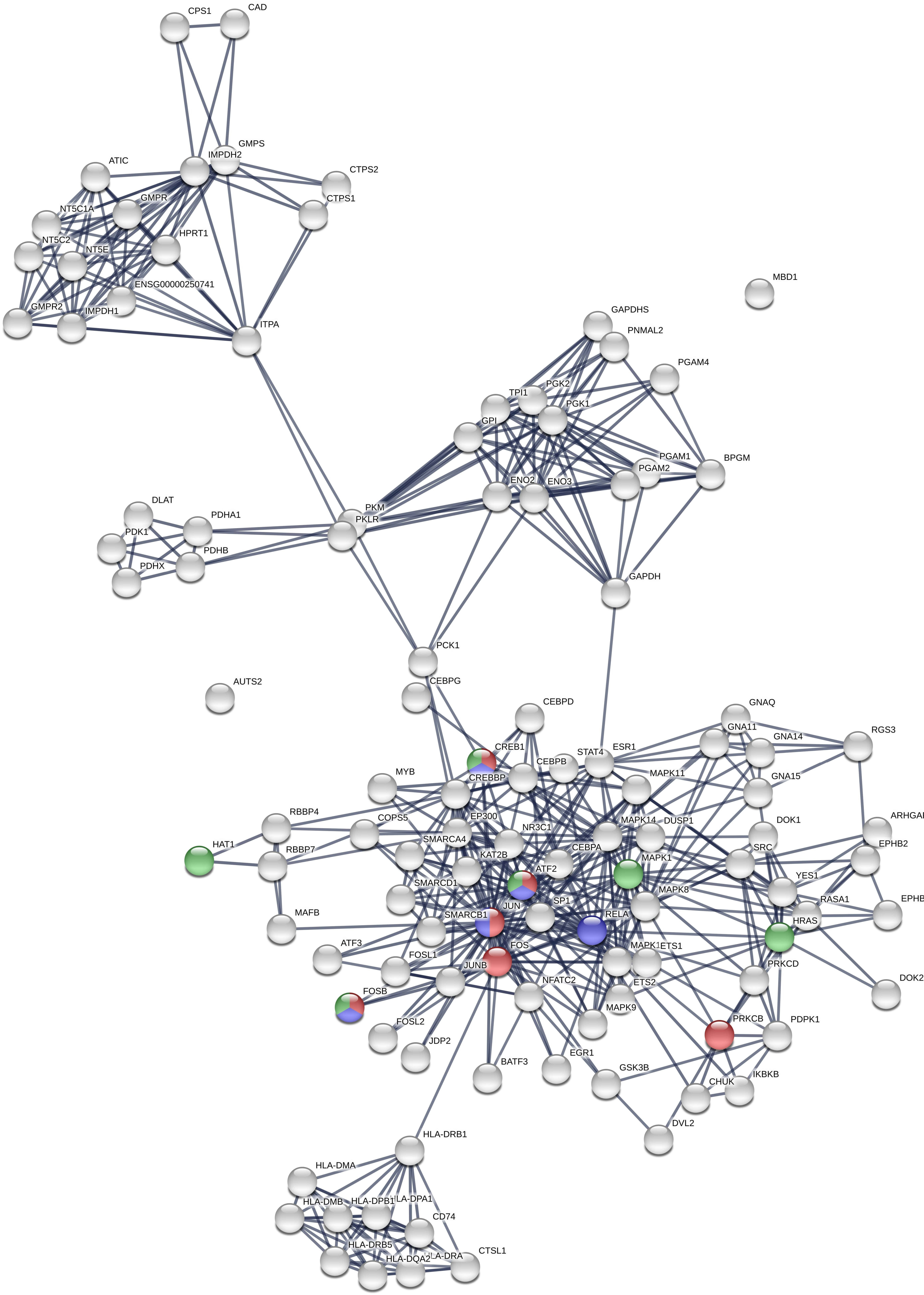

CPS1
CAD
GMPS
IMPDH2
ATIC
CTPS2
CTPS1
GMPR
NT5C1A
HPRT1
NT5C2
NT5E
ENSG00000250741
GMPR2
IMPDH1
ITPA
MBD1
GAPDHS
PNMAL2
PGAM4
TPI1
PGK2
PGK1
GPI
PGAM1
PGAM2
BPGM
ENO2
ENO3
DLAT
PDHA1
PDK1
PDHB
PDHX
PKM
PKLR
GAPDH
PCK1
AUTS2
CEBPG
CEBPD
GNAQ
GNA11
GNA14
RGS3
CREB1
CEBPB
STAT4
ESR1
MYB
CREBBP
MAPK11
GNA15
RBBP4
COPS5
EP300
NR3C1
MAPK14
DUSP1
DOK1
HAT1
RBBP7
SMARCA4
KAT2B
CEBPA
MAPK1
SRC
EPHB2
ATF2
YES1
SMARCD1
MAPK8
RASA1
MAFB
SMARCB1
JUN
SP1
RELA
HRAS
ATF3
FOS
MAPK1
ETS1
FOSL1
JUNB
PRKCD
NFATC2
ETS2
DOK2
FOSB
MAPK9
FOSL2
PRKCB
PDPK1
JDP2
BATF3
EGR1
GSK3B
CHUK
IKBKB
DVL2
HLA-DRB1
HLA-DMA
HLA-DMB
HLA-DPB1
CD74
HLA-DRB5
HLA-DQA2
CTSL1

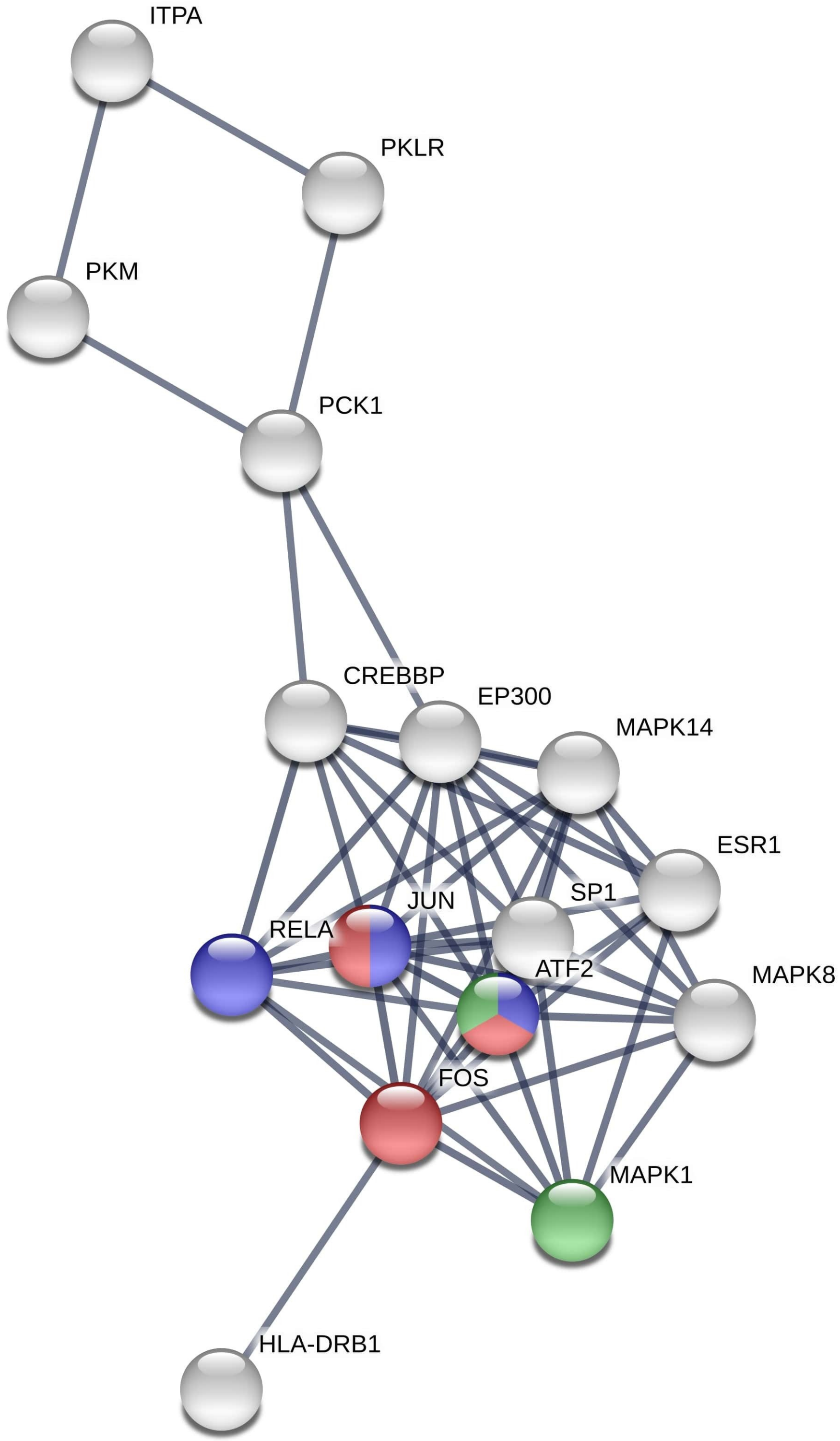
ITPA
PKLR
PKM
PCK1
CREBBP
EP300
MAPK14
ESR1
SP1
JUN
RELA
ATF2
MAPK8
FOS
MAPK1
HLA-DRB1

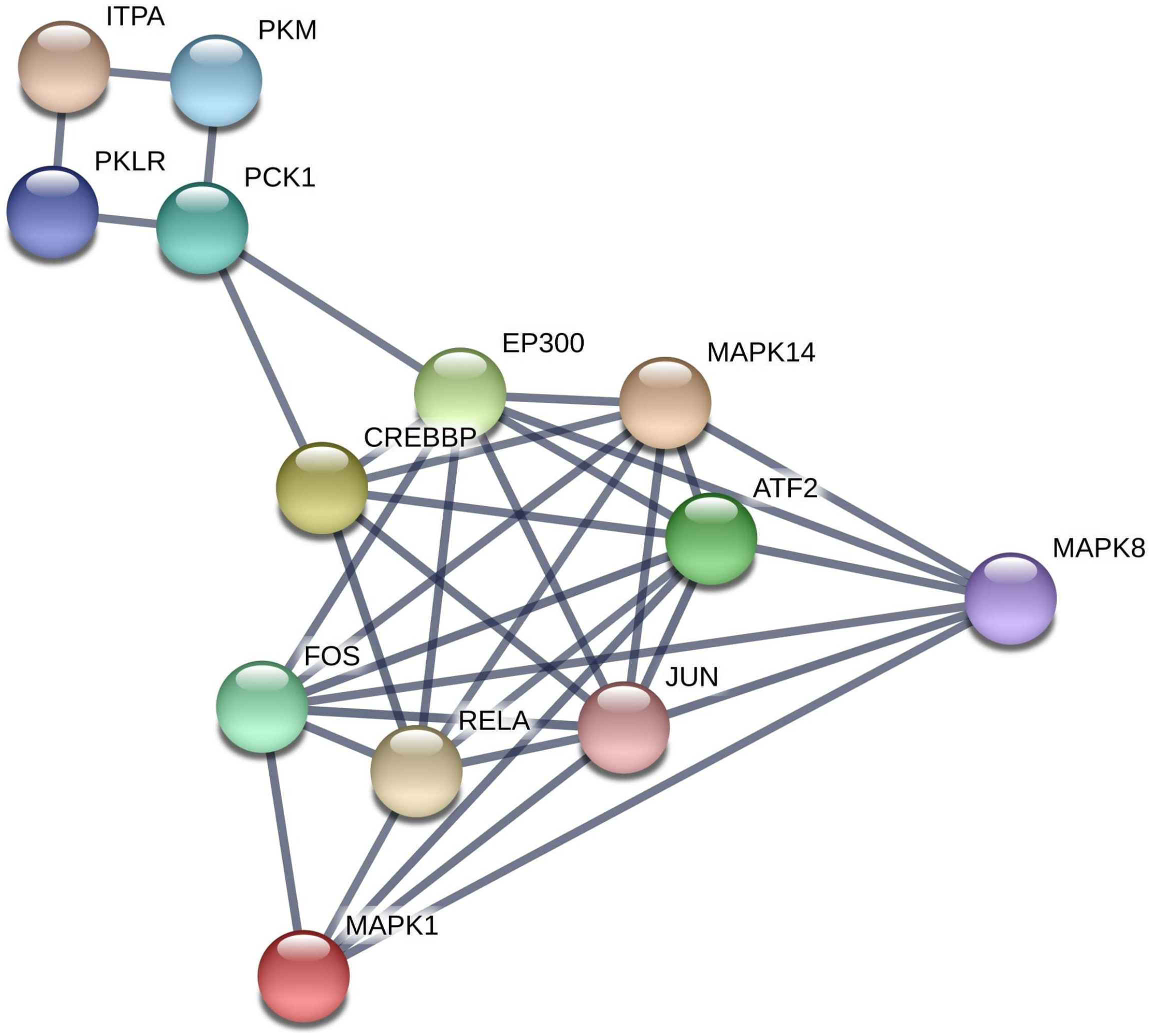
ITPA
PKM
PKLR
PCK1
EP300
MAPK14
CREBBP
ATF2
MAPK8
FOS
JUN
RELA
MAPK1

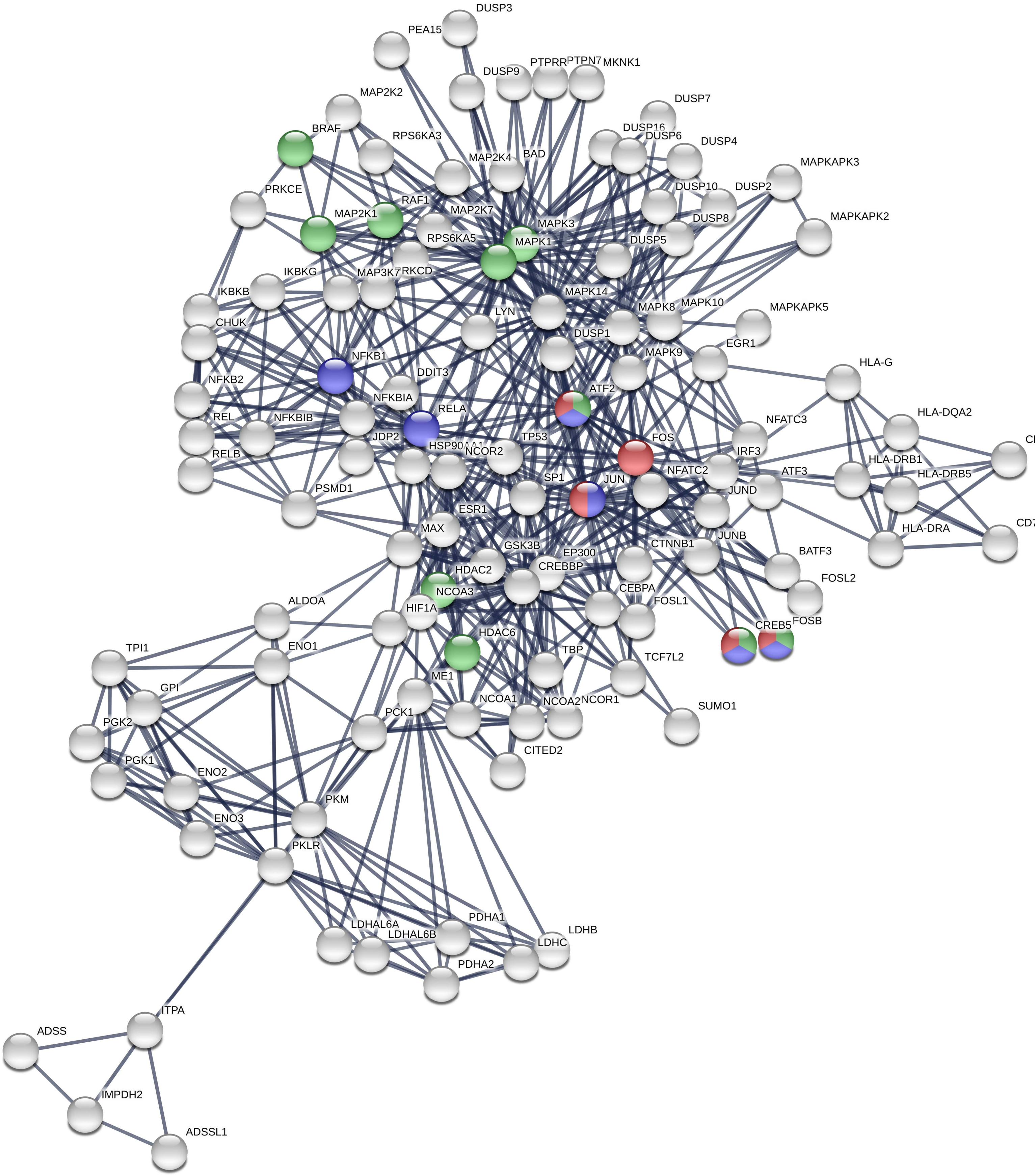

DUSP3
PEA15
DUSP9
PTPRR
PTPN7
MKNK1
MAP2K2
DUSP7
BRAF
RPS6KA3
DUSP16
DUSP6
DUSP4
MAP2K4
BAD
MAPKAPK3
PRKCE
RAF1
MAP2K7
DUSP10
DUSP2
MAP2K1
MAPK3
DUSP8
MAPKAPK2
RPS6KA5
MAPK1
DUSP5
IKBKG
MAP3K7
IKBKB
MAPK14
MAPK8
MAPK10
MAPKAPK5
LYN
CHUK
DUSP1
EGR1
NFKB1
MAPK9
HLA-G
DDIT3
NFKB2
ATF2
NFKBIA
RELA
NFATC3
HLA-DQA2
REL
NFKBIB
FOS
JDP2
TP53
CD4
NCOR2
IRF3
RELB
NFATC2
ATF3
HLA-DRB1
HLA-DRB5
SP1
JUN
PSMD1
JUND
ESR1
MAX
JUNB
HLA-DRA
CD74
GSK3B
CTNNB1
EP300
BATF3
HDAC2
CREBBP
FOSL2
NCOA3
CEBPA
ALDOA
HIF1A
FOSL1
CREB5
FOSB
TPI1
ENO1
HDAC6
TBP
TCF7L2
GPI
ME1
NCOA1
NCOA2
NCOR1
SUMO1
PGK2
PCK1
CITED2
PGK1
ENO2
PKM
ENO3
PKLR
PDHA1
LDHAL6A
LDHB
LDHAL6B
LDHC
PDHA2
ITPA
ADSS
IMPDH2
ADSSL1

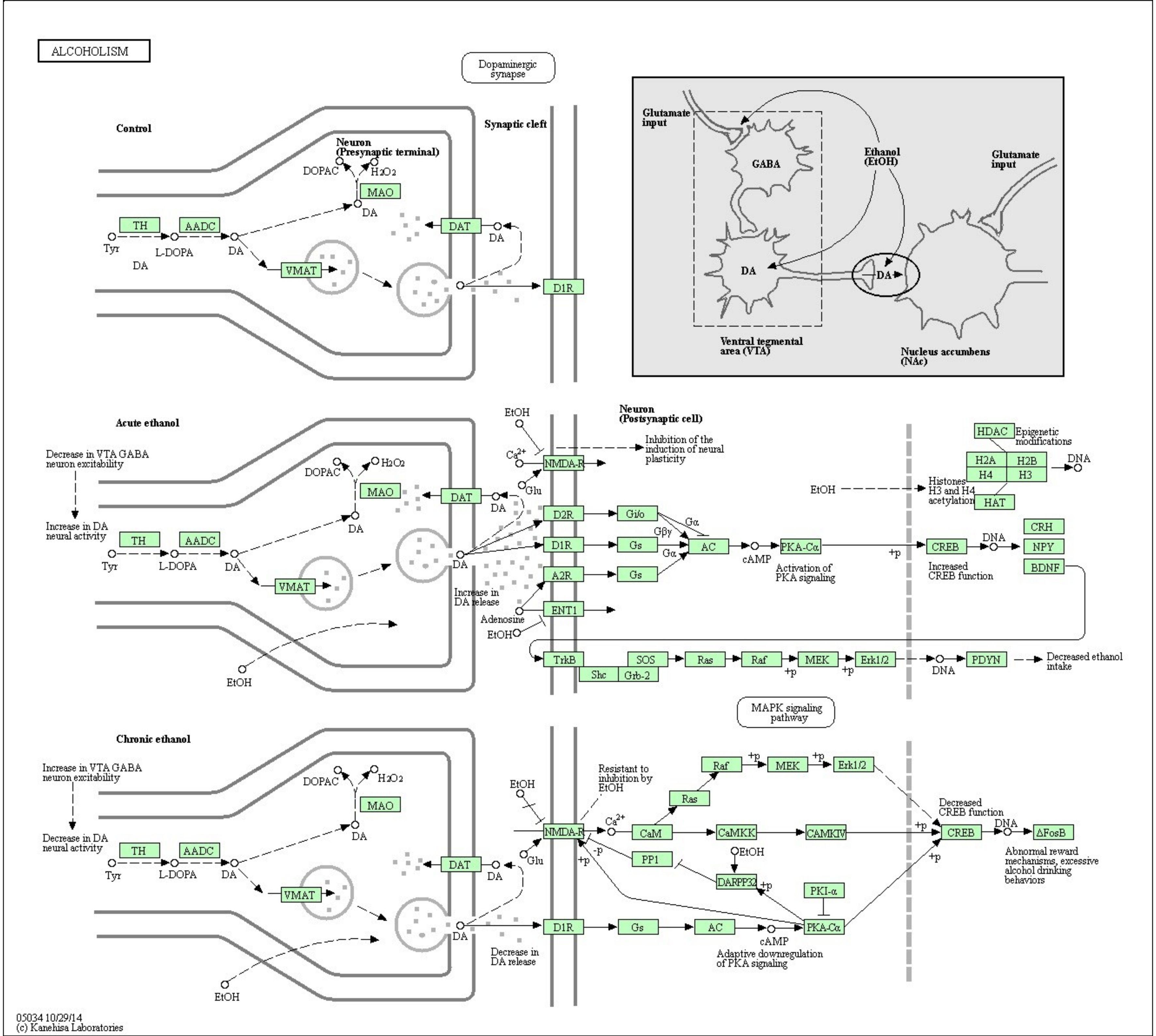
ALCOHOLISM
Dopaminergic synapse
Control
Neuron (Presynaptic terminal)
Synaptic cleft
DOPAC
H2O2
MAO
DA
TH
AADC
DAT
Tyr
L-DOPA
VMAT
D1R
Glutamate input
GABA
Ethanol (EtOH)
Glutamate input
DA
DA
Ventral tegmental area (VTA)
Nucleus accumbens (NAc)
Acute ethanol
EtOH
Neuron (Postsynaptic cell)
Decrease in VTA GABA neuron excitability
Inhibition of the induction of neural plasticity
Ca2+
NMDA-R
Glu
HDAC
Epigenetic modifications
H2A
H2B
H4
H3
DNA
EtOH
Histones H3 and H4 acetylation
HAT
Increase in DA neural activity
D2R
Gi/o
Gα
Gβγ
AC
cAMP
PKA-Cα
+p
CREB
CRH
NPY
BDNF
Gs
A2R
Activation of PKA signaling
Increased CREB function
Increase in DA release
ENT1
Adenosine
TrkB
Shc
SOS
Grb-2
Ras
Raf
MEK
Erk1/2
PDYN
Decreased ethanol intake
MAPK signaling pathway
Chronic ethanol
Increase in VTA GABA neuron excitability
Resistant to inhibition by EtOH
Decrease in DA neural activity
CaM
CaMKK
CAMKIV
Decreased CREB function
ΔFosB
PP1
-p
+p
DARPP32
PKI-α
Abnormal reward mechanisms, excessive alcohol drinking behaviors
Decrease in DA release
Adaptive downregulation of PKA signaling
05034 10/29/14
(c) Kanehisa Laboratories

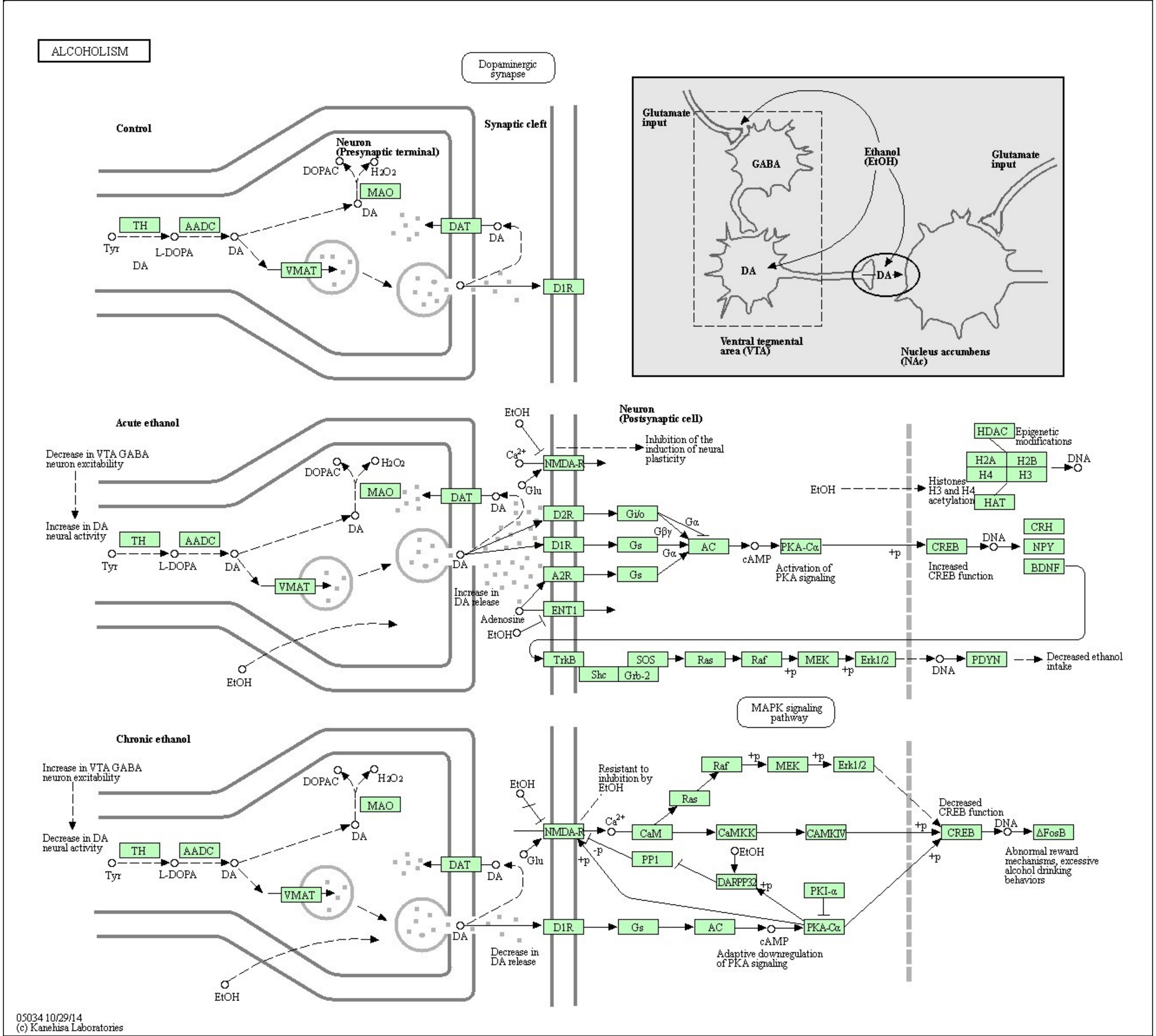
ALCOHOLISM
Dopaminergic synapse
Control
Neuron (Presynaptic terminal)
Synaptic cleft
DOPAC
H2O2
MAO
DA
TH
AADC
Tyr
L-DOPA
DAT
VMAT
D1R
Glutamate input
GABA
Ethanol (EtOH)
Glutamate input
DA
Ventral tegmental area (VTA)
Nucleus accumbens (NAc)
Acute ethanol
Decrease in VTA GABA neuron excitability
Increase in DA neural activity
EtOH
Neuron (Postsynaptic cell)
Ca2+
NMDA-R
Glu
Inhibition of the induction of neural plasticity
D2R
Gi/o
Gα
Gβγ
Gs
AC
cAMP
PKA-Cα
Activation of PKA signaling
A2R
ENT1
Adenosine
Increase in DA release
HDAC
Epigenetic modifications
H2A
H2B
H4
H3
HAT
DNA
Histones H3 and H4 acetylation
+p
CREB
Increased CREB function
CRH
NPY
BDNF
TrkB
Shc
SOS
Grb-2
Ras
Raf
MEK
Erk1/2
PDYN
Decreased ethanol intake
MAPK signaling pathway
Chronic ethanol
Increase in VTA GABA neuron excitability
Decrease in DA neural activity
Resistant to inhibition by EtOH
CaM
CaMKK
CAMKIV
PP1
DARPP32
PKI-α
-p
Decreased CREB function
ΔFosB
Abnormal reward mechanisms, excessive alcohol drinking behaviors
Adaptive downregulation of PKA signaling
Decrease in DA release
05034 10/29/14
(c) Kanehisa Laboratories

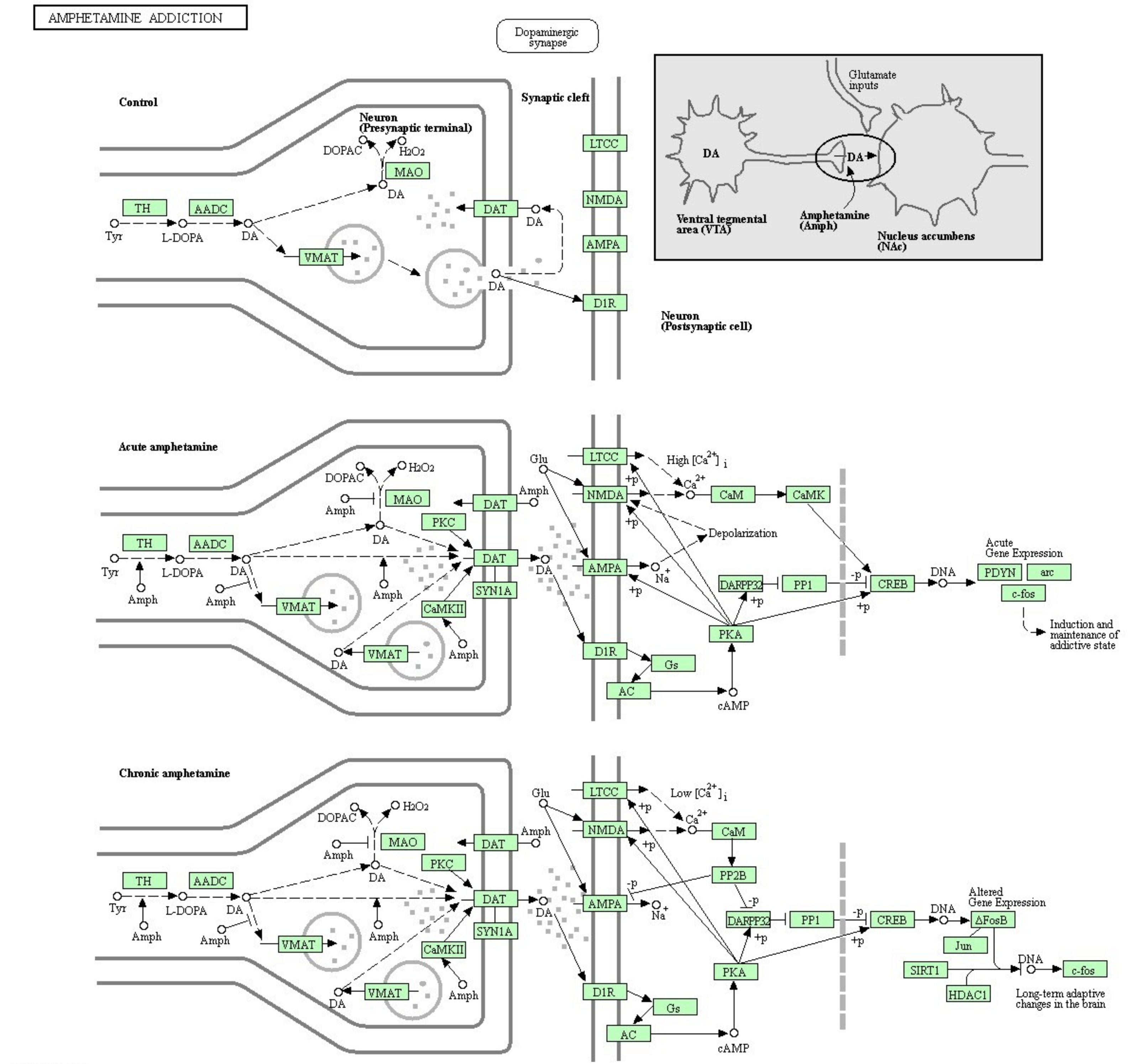

05031 10/23/15
(c) Kanehisa Laboratories
